# Simultaneous Removal of Pb^2+^ and Zn^2+^ Heavy Metals Using Fly Ash Na-X Zeolite and Its Carbon Na-X(C) Composite

**DOI:** 10.3390/ma14112832

**Published:** 2021-05-25

**Authors:** Rafał Panek, Magdalena Medykowska, Małgorzata Wiśniewska, Katarzyna Szewczuk-Karpisz, Katarzyna Jędruchniewicz, Małgorzata Franus

**Affiliations:** 1 Faculty of Civil Engineering and Architecture, Lublin University of Technology, Nadbystrzycka 40, 20-618 Lublin, Poland; m.franus@pollub.pl; 2 Department of Radiochemistry and Environmental Chemistry, Institute of Chemical Sciences, Faculty of Chemistry, Maria Curie-Sklodowska University in Lublin, M. Curie-Sklodowska Sq. 3, 20-031 Lublin, Poland; wisniewska@hektor.umcs.lublin.pl (M.W.); k.jedruchniewicz@poczta.umcs.lublin.pl (K.J.); 3 Institute of Agrophysics, Polish Academy of Sciences, Doświadczalna 4, 20-290 Lublin, Poland; k.szewczuk-karpisz@ipan.lublin.pl

**Keywords:** fly ash, synthetic zeolites, zeolite-carbon composites, adsorbents of heavy metals, competitive adsorption, simultaneous removal, adsorption kinetics

## Abstract

Pure zeolite (Na-X) and a zeolite–carbon composite (Na-X(C)) were investigated as adsorbents of heavy metals—Pb^2+^ and Zn^2+^ from an aqueous solution. These materials were synthesized from fly ash—a waste from conventional hard coal combustion. Both solids were characterized using XRD, SEM-EDS, nitrogen adsorption/desorption, particle size and elemental composition analyses. The adsorption study was performed at pH 5 in the systems containing one or two adsorbates simultaneously. The obtained results showed that the pure zeolite was characterized by a more developed surface area (728 m^2^/g) than its carbon composite (272 m^2^/g), and the mean pore diameters were equal to 1.73 and 2.56 nm, respectively. The pure Na-X zeolite showed better adsorption properties towards heavy metals than its Na-X(C) composite, and Zn^2+^ adsorbed amounts were significantly higher than the Pb^2+^ ones (the highest experimental adsorption levels were: for Zn^2+^—656 and 600 mg/g, and for Pb^2+^—575 and 314 mg/g, on the Na-X and Na-X(C) surfaces, respectively). The zinc ions are exchanged with the cations inside the zeolite materials structure more effectively than lead ions with a considerably larger size. In the mixed systems, the competition between both heavy metals for access to the active sites on the adsorbent surface leads to the noticeable reduction in their adsorbed amounts. Moreover, the hydrochloric acid was a better desorbing agent for both heavy metals, especially Pb^2+^ one (desorption reached 78%), than sodium base (maximal desorption 25%).

## 1. Introduction

Zeolites are hydrated aluminosilicates of metals from the I and II group, formed in hydrothermal processes of rock transformation in the environment or synthesized by chemical methods. These materials are characterized by unique structures. They are composed of silicon and aluminum tetrahedrons, called Primary Building Units (PBU). The tetrahedrons are connected by oxygen atoms, which create larger repeating geometric forms called polyhedrons. They are signed as Secondary Building Units (SBU). As a result of a combination of the units, three-dimensional zeolite structures such as cubes, hexagonal pyramids or cuboctahedrons are formed. Furthermore, the specific distribution of tetrahedrons creates an internal system of channels filled, for example, with water molecules (called zeolite water). Zeolites are extremely diverse in their chemical composition. Usually, the negative charge of the crystal lattice, easy exchange of extra-network cations, homogeneous micropore size and satisfactory thermal and hydrothermal stability are features typical for the described materials [1,2]. Zeolites exhibit well-developed specific surface, high sorption capacity and high ion exchange [3,4,5,6]. Due to this fact, they are called molecular sieves [1]. They can adsorb not only cations, for instance, heavy metal ions, but also organic substances or anions from aqueous solutions. The properties of natural zeolites can be improved by their modification using ion exchange, acid treatment or functionalization with surfactants.

Heavy metals have a negative impact on the natural environment and living organisms. They are often toxic, cancerogenic and can damage the immune and nervous system. Therefore, it is important to monitor and, if possible, remove this type of contamination from water, sewage and soil. Currently, scientists are focusing more and more on the use of sorbents obtained from various types of waste for heavy metals removal. Shahrokhi-Shahraki et al. confirmed that the activated carbon from pulverized waste tire shows a better sorption performance towards Pb^2+^, Cu^2+^ and Zn^2+^ than its commercial counterpart [7]. Other sorbents used for the removal of heavy metals are zeolites from the sodalite and gismodite groups obtained from fly ash [8]. Fly ash-derived zeolites have also been used in simultaneous sorption in mixed systems (even five different heavy metals) [9,10]. Similar or even better sorption results were also obtained in another study, in which fly ash-derived zeolites from the faujasite group were used for heavy metals removal (Pb^2+^, Cu^2+^, Cd^2+^, Zn^2+^, Co^2+^) in an aqueous solution [11].

In this paper, zeolite (Na-X) and novel zeolite–carbon composite (Na-X(C)) as adsorbents of heavy metal ions are presented. The sorption capacity of the prepared materials relative to two heavy metals, Pb^2+^ and Zn^2+^, was investigated. Other scientists have also tested the adsorption abilities of modified and non-modified zeolites relative to the selected metal ions. However, they did not observe spectacularly high adsorption capacities. Kragović et al. [12] investigated natural zeolite (NZA) and zeolite modified via alginate (FRA) and noted that the adsorbed amount on NZA was 102 mg/g and 133 mg/g for FRA. Kim et al. [13] used nanoscale zero-valent iron to obtain the zeolite composite (Z-nZVI) and stated that the Pb^2+^ adsorbed amount on this material was equal to 96.2 mg/g. Li et al. [14] also examined zeolite-supported nanoscale zero-valent iron and stated that the adsorption capacity of this material relative to Pb^2+^ ions was 85.37 mg/g. Wang et al. [15] investigated the zeolitic imidazolate framework grown on graphene oxide (ZIF-8@GO) and obtained maximum adsorption capacity for Pb^2+^ ions equal to 356 mg/g. Abdelrahman et al. [16] used rice husks and aluminum can waste to obtain geopolymer–zeolite products (G3). The adsorbed amount of Zn^2+^ ions on this material was 131.93 mg/g. Wang et al. [17] synthesized SiO_2_ encapsulated natural zeolite for the removal of heavy metal ions. The amount of adsorbed Pb^2+^ ions on this solid was 186 mg/g, whereas, for Zn^2+^ ions, it was 9 mg/g. The zeolite–carbon composites have not been reported yet. Thus, the authors decided to examine precisely these materials.

The adsorbents used in the experiments were manufactured in the process of hydrothermal conversion of high-carbon fly ash (HCFA) [18,19,20]. This byproduct is accounted for 65–95% of total waste produced in the energy sector. Therefore, both adsorbents were produced from waste in one cycle of the HCFA treatment process—Na-X(C) composite directly from HCFA and Na-X from the secondary waste generated during previous synthesis (silica-rich solution). The whole process was conducted on a semi-technical scale, which can be applied in industries in the near future. Owing to these facts, the solids used in the study have an undoubtfully innovative nature. In order to achieve the full characterization of zeolite and zeolite–carbon composite, various methods, such as SEM-EDS, XRD, nitrogen adsorption/desorption, elemental analysis, potentiometric titration, zeta potential measurement, etc., were applied. The adsorption of heavy metal ions was investigated in the single and mixed systems, i.e., containing one or two heavy metal types at the same time. This allowed for recreating the natural conditions and show the influence of one adsorbate on the adsorption of another one. Contamination in soils by heavy metal ions is a huge environmental problem originating from anthropogenic activities, such as mining, the chemical industry, agriculture or atmospheric deposition. Especially near industrial bases, there is a strong possibility of dangerous pollution posing a risk to organisms for a long time [21,22,23]. The performed experiments allowed to state whether selected materials allow for highly effective cleaning of soil and water.

## 2. Materials and Methods

### 2.1. Adsorbent Preparation

In the first stage of the experiment, the zeolite–carbon composite (Na-X(C)) was obtained. The starting material was fly ash from the Janikowo thermal power plant (Janikowo, Poland) produced as a result of conventional hard coal combustion. The carbon–zeolite composite was created in the hydrothermal reaction of fly ash (25 kg) with a 3 M aqueous sodium hydroxide solution (90 L) on the technological line for the synthesis of ash zeolites for 48 h in 80 °C [19]. On the other hand, Na-X was obtained from a waste solution rich in silicon and aluminum (formed during the synthesis of Na-X(C)) according to the procedure contained by Panek et al. [18]. Briefly, this waste solution was mixed with a sodium hydroxide solution containing Al foil and then subjected to a hydrothermal reaction. Mineralogical, structural and textural characteristics were conducted on both sorbents.

### 2.2. Solids Characteristics

Textural parameters were measured on an ASAP 2020 apparatus (Micromeritics Instrument Corporation, Norcross, GA, USA) using low-temperature nitrogen adsorption/desorption isotherms at a liquid nitrogen temperature of −194.85 °C over a range of relative pressures p/p_0_ ranging from 1.5 × 10^−7^ to 0.99. The shape analysis of nitrogen vapor adsorption isotherms was characterized based on the IUPAC classification [24,25]. Samples were degassed twice: (1) at 300 °C for 12 h under reduced pressure, (2) 4 h at 300 °C (prior to analysis). The specific surface area (S_BET_) of adsorbents was determined using the BET equation. The micropore volume (V_micro_) and area (S_micro_) were determined using the ‘t-plot’ method. In turn, the pore size distribution in the adsorbent was calculated using the BJH method.

The elemental composition was determined by semi-quantitative energy dispersive X-ray fluorescence on an Epsilon 3 spectrometer (Panalytical, Eindhoven, The Netherlands). The apparatus was equipped with a Rh 9 W, 50 kV, 1 mA X-ray lamp, a 4096 channel spectrum analyzer and a high-resolution solid-state SDD detector cooled by a Peltier cell. Obtained results included the LOI.

The particle size was investigated based on laser diffraction phenomena on a Mastersizer 3000 instrument (Malvern Panalytical, Malvern, UK). The test was conducted in an aqueous environment in the HYDRO EV attachment. The Mie theory was used during measurements in the measurement range (0.01 µm to 2 mm).

A morphological analysis in the microarray of the studied materials was carried out on a Quanta 250 FEG scanning electron microscope (FEI, Hillsboro, OR, USA) equipped with an EDS attachment from EDAX. The experiments were conducted on samples sprayed with a conductive carbon layer at an accelerating voltage of 15 keV.

The mineral composition of sorbents was determined by X-ray diffraction on XPert Pro MPD (Panalytical, Eindhoven, The Netherlands). A copper lamp (CuK_α_ = 1.54178 Å) was used as the emission source. The test was conducted over an angular range of 5–65° 2Θ with a step equal to 0.02° 2θ lasting 5 s. The X’Pert Highscore software was used to process the diffraction data. The identification of mineral phases was based on the PDF-2 release 2010 database formalized by JCPDS-ICDD.

The surface functional groups of zeolite and zeolite–carbon composite were determined using Fourier transform infrared spectroscopy (FTIR spectrometer, Nicolet 8700A, Thermo Scientific, Waltham, MA, USA). The solids were analyzed as pellets with KBr.

### 2.3. Adsorption/Desorption Measurements

The adsorbed amounts of heavy metal ions on the Na-X and Na-X(C) surfaces were determined using the static method, based on the decrease in the adsorbate concentration in the solution after the adsorption process [26]. The samples (10 cm^3^) were prepared using 0.003 g of the solid, supporting electrolyte (0.001 M NaCl) and heavy metal ion with the concentration in the range of 10–200 ppm. In the mixed systems, the concentration of ions was equal to 100 ppm. The bulk solutions of heavy metal (Pb(NO_3_)_2_ and Zn(NO_3_)_2_) had a concentration of 1000 ppm. The appropriate concentration of the adsorbates was obtained by diluting them with demineralized water. The adsorption process was carried out for 3 h, at pH 5, under continuous shaking conditions (100 rpm, Unimax 1010, Heidolph, Schwabach, Germany). The pH value was adjusted in the samples using 0.1 M NaOH or 0.1M HCl and pHmeter Beckman (Pasadena, CA, USA) when adsorbates were present in the suspensions. The used HCl/NaOH volumes were maximum 0.005 cm^3^. After the adsorption completion, the solid was separated by centrifugation (8000 rpm, 310 b, Mechanika Precyzyjna, Poland). The heavy metal ions concentration in the supernatants was determined using inductively coupled plasma-optical emission spectrometry (Thermo Scientific iCAP™ 7200 ICP-OES analyzer, Waltham, MA, USA). The adsorption isotherm data were fitted to the Langmuir (Equation (1)) and Freundlich (Equation (2)) models [26,27]:

(1) $$q_{e}=\frac{q_{m}K_{L}C_{e}}{1+K_{L}C_{e}}$$

(2) $$q_{e}=K_{F}C_{e}^{1/n}$$

where K_F_ and K_L_—the Freundlich (mg/g (mg/dm^3^)^−1/nF^) and Langmuir (dm^3^/mg) parameters, q_e_—the equilibrium adsorption capacity (mg/g), C_e_—the equilibrium liquid phase concentration (mg/L), q_m_—the maximum adsorption capacity in Langmuir model (mg/g), n—the Freundlich constant related to adsorption intensity.

The samples for kinetics study were prepared using 100 ppm of heavy metal ions, supporting electrolyte (0.001 M NaCl) and 0.003 g of Na-X or Na-X(C). The adsorption was carried out for a specific time (10–200 min), at pH 5, under continuous shaking conditions. After filtration of the samples using paper filters (389, Ahlstrom Munktell, Helsinki, Finland), the concentration of heavy metal ions in the clear solutions was determined using ICP-OES. The paper filters were used to isolate the solid more quickly, which is necessary for correct kinetic measurements. The obtained results were modelled using pseudo-first-order (PFO, Equation (3)) and pseudo-second-order equations (PSO, Equation (4)) [28,29]:

(3) $$\frac{{\mathrm{dq}}_{t}}{\mathrm{dt}}=k_{1}\left(q_{e}-q_{t}\right)$$

(4) $$\frac{{\mathrm{dq}}_{t}}{\mathrm{dt}}=k_{2}{\left(q_{e}-q_{t}\right)}^{2}$$

where q_e_—the adsorbed amount at equilibrium (mg/g), q_t_—the adsorbed amount after time ‘t’ (mg/g), k_1_ (1/min) and k_2_ (g/mg·min)—the equilibrium rate constants.

Desorption rate was determined using the solids obtained after the adsorption, separated from the samples of initial concentration of heavy metal ions equal to 100 ppm. The solids were transferred to 10 mL of 0.1 M HCl or 0.1 M NaCl solutions and the desorption process was performed for 1 h, under continuous shaking (100 rpm, Unimax 1010, Heidolph, Schwabach, Germany). After that, the separation of the solids from the solutions was repeated and the ion concentration was determined by ICP-OES.

The one result of the adsorbed/desorbed amount was the average of three repetitions. The average error did not exceed 5%.

### 2.4. Electrokinetic Parameters Determination

The surface charge density (σ_0_) of the zeolite and its carbon-based composite, with and without adsorbates, was determined by potentiometric titration. The σ_0_ values as a function of the solution pH were calculated using the computer program “titr_v3”, on the basis of the difference in the volume of the titrant added to the suspension and the supporting electrolyte solution providing a specific pH value [30]. The titration was performed using a set that consisted of: teflon vessel connected to a RE 204 thermostat (Lauda, Lauda-Königshofen, Germany), automated Dosimat 765 microburette (Metrohm, Herisau, Switzerland), glass and calomel electrodes (Beckman Instruments, Pasadena, CA, USA), PHM 240 pH meter (Radiometer, Copenhagen, Denmark) and a computer. The examined samples were titrated with 0.1 M NaCl solution, in the pH range from 3 to 11. The solutions were prepared by adding 0.03 g Na-X or 0.075 g Na-X(C) to 50 cm^3^ of supporting electrolyte (0.001 M NaCl). At first, only the electrolyte was titrated, then the suspensions, with and without adsorbates (10 ppm), was examined.

The measurements of the electrophoretic mobility (u_e_) enabled the calculation of the zeta potential (ζ) of the particles of zeolite and its carbon composite [31]. The experiments were carried out using the Zetameter Nano ZS (Malvern Instruments, Cambridge, UK). The suspensions were prepared by adding 0.01 g of Na-X or Na-X(C) to 200 cm^3^ of the supporting electrolyte solution (0.001 M NaCl), with and without one or two heavy metal ions (10 ppm). After a 3-min sonication, the suspension was divided into several parts and a specific pH value was adjusted in each of them (in the range from 3 to 10).

## 3. Results and Discussion

### 3.1. Physicochemical Properties of Adsorbents

The textural parameters of zeolite and zeolite–carbon composite are summarized in Table 1.

The obtained textural parameters indicated that zeolite Na-X was characterized by a more developed surface than its carbon composite. Na-X has a much higher specific surface area (728 m^2^/g) and greater content of micropores in its structure (about 87%). In the case of the Na-X(C) composite, the results are in agreement with those obtained by other authors [19]. For Na-X, the S_BET_ value is even higher than for commercial 13X or those obtained from chemical reagents [32]. It should be mentioned that when zeolite was obtained directly from waste, e.g., from fly ash, the S_BET_ value is much lower ranged from 236 to 257 m^2^/g [33,34].

The chemical composition of zeolite–carbon Na-X(C) composite and Na-X zeolite is presented in Table 2.

The chemical composition of the Na-X(C) composite and Na-X zeolite is dominated by silicon 28.19% and 41.12% and aluminum by 14.37% and 23.41%, respectively. Moreover, there are negligible amounts of CaO (2.21–7.48%), Fe_2_O_3_ (1.31–9.33%) and Na_2_O (3.53–7.32%). It is worth mentioning that Si/Al molar ratios for Na-X and Na-X(C) was 1.49 and 1.66, respectively. It can be found that Na-X(C) has high Si/Al molar ratio. The Si/Al molar ratio values highly correspond to the results obtained by other researchers [18,19]. Boycheva, with coworkers, determined a similar Si/Al molar ratio (1.37–1.60) for Na-X zeolite synthesized from coal fly ash by hydrothermal reaction and with preliminary fusion step [35].

**Figure 1:**
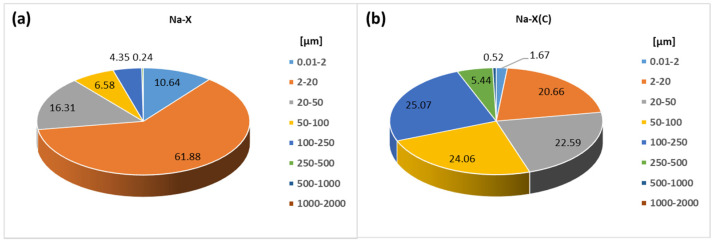
Particle size distribution (*v/v* %) of (**a**) Na-X and (**b**) Na-X(C) materials.

The particle size distributions for the tested materials are presented in the diagrams in Figure 1a,b.

In the case of zeolite–carbon composite, four fractions dominate: 2–20 µm, 20–50 µm, 50–100 µm and 100–250 µm and account for 92.38%. Moreover, in the case of the carbon composite, there is a small fraction volume of 250–500 µm (5.44%), which corresponds to results obtained by other researchers [19]. Pure Na-X zeolite has a very fine particle size of 2–20 µm (61.88%), which is very similar to the value received by Panek et al. [18]. This material is also characterized by the highest content of the finest fraction 0.01–2 µm, constituting 10.64%, and a small content of larger fractions: 16.31% for 20–50 µm, 6.58% for 50–100 µm and 4.35% for 100–250 µm.

**Figure 2:**
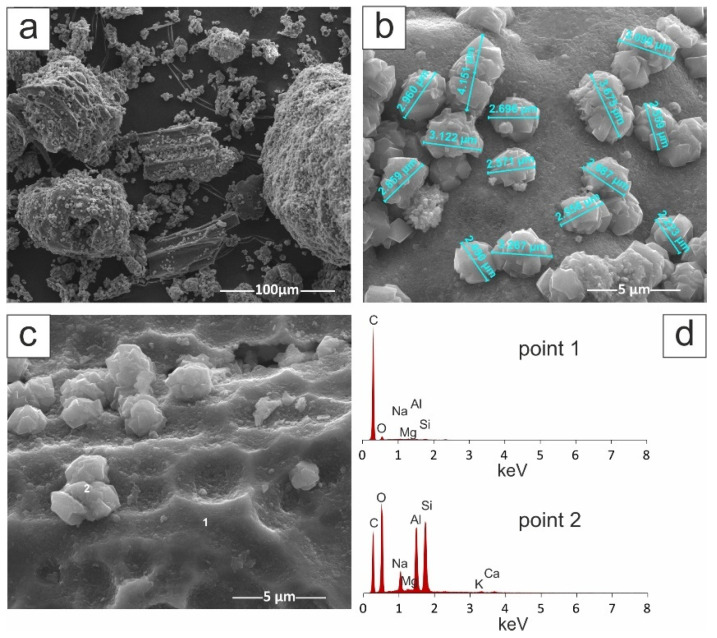
SEM images of Na-X(C) sample: (**a**) 1000× magnification, (**b**) 16,000× magnification, (**c**) 16,000× magnification with EDS chemical analysis (**d**).

Figure 2 shows the morphology of the zeolite–carbon Na-X(C) composite.

**Figure 3:**
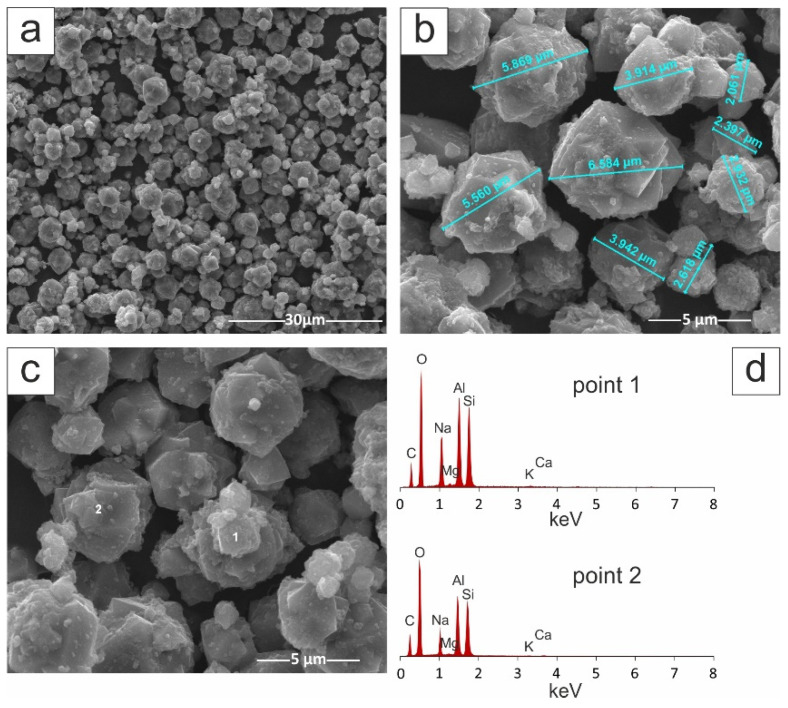
SEM images of Na-X sample: (**a**) 4000× magnification, (**b**) 16,000× magnification, (**c**) 16,000× magnification with EDS chemical analysis (**d**).

Zeolite crystals are very well formed and characterized by an isomeric structure with diameters ranging from 2 µm to 5 µm. These crystals are formed in the carbon ash zone, as well as on fragments of aluminosilicate glaze. They have the character of single crystals or mutually interspersed aggregates. Figure 2 also shows the chemical EDS analysis performed on the carbon fragment (point 1) and the zeolite crystal (point 2). In the first case, a chemical analysis showed the presence of C, and in the second case, the dominant elements were Al, Si, Na, Mg and O. The C presence in the sample was dictated by the sample preparation. Figure 3 shows the morphology of pure Na-X zeolite (no ash residue).

The zeolite crystals are very well formed with a size from 2 µm to 5 µm. Figure 3 also shows the EDS analysis performed on zeolite crystals (points 1 and 2). In both cases, the dominant elements were Si, Al, Na and O and small amounts of Ca, K and Mg. The presence of C in the sample was associated with the sample preparation.

**Figure 4:**
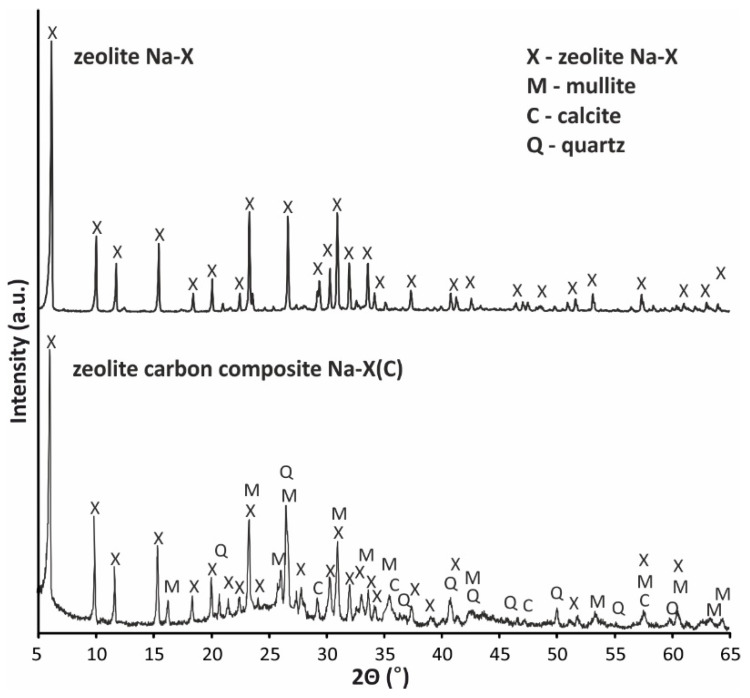
Phase analysis of zeolite–carbon Na-X(C) composite and Na-X zeolite.

Diffractograms of the phase composition of Na-X(C) composite and pure Na-X zeolite are shown in Figure 4.

The diffractograms of the Na-X(C) composite and Na-X zeolite indicate that only the pure Na-X zeolite sample obtained from the filtrate has a monomineral character. The presence of the zeolite phase was characterized by the following reflections d_hkl_ = 14.42; 8.83; 5.71; 3.83; 3.35; 2.90 Å (reference code 00-038-0237). In addition, on the diffractograms, a higher background level (between 15 and 35° 2θ) was not observed, which is normal for zeolites obtained directly from fly ash being a proof of amorphous aluminosilicate glaze presence [36]. On the other hand, on the diffractogram of composite phases related to unreacted ash residue in the form of aluminosilicate glaze, mullite (d_hkl_ = 5.38; 3.40; 2.79; 2.59 Å; reference code 01-089-2645), quartz (d_hkl_ = 4.25; 3.34; 2.45; 2.28 Å; reference code 01-073-8320) and calcite (d_hkl_ = 3.03; 2.49; 2.09; 1.88 Å; reference code 01-086-2334) were observed. Such phase characteristics is typical for fly ash-derived zeolites [37].

**Figure 5:**
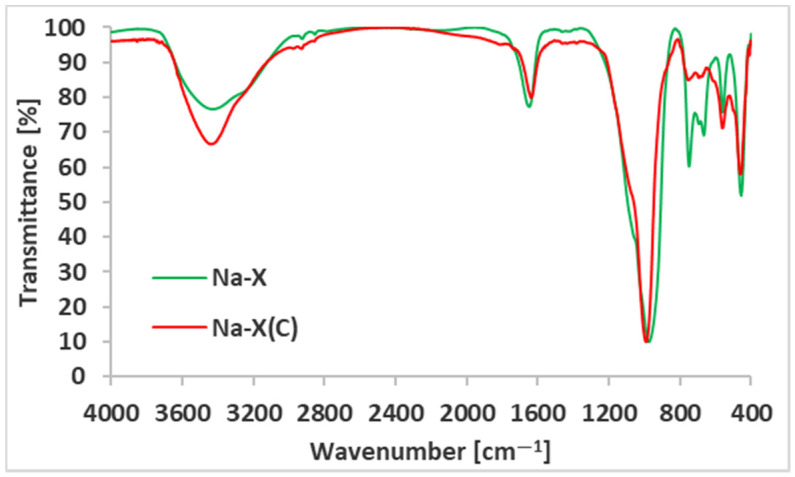
FTIR spectra of Na-X and Na-X(C).

The FTIR spectra of zeolite and zeolite–carbon composite are presented in Figure 5.

The FTIR spectra of zeolite and zeolite–carbon composite contained the following bands at: 3386.39 cm^−1^ and 1636.30 cm^−1^ (corresponding with stretching and bending of water molecules adsorbed on solids), 979.66 cm^−1^ (Si-O-Al anti-symmetric stretching vibration of T-O bonds, T—tetrahedrally bonded Si or Al), 550.09 cm^−1^ (symmetric stretching of double six membered rings of T-O-T), 449.33 cm^−1^ (symmetric T-O bending). The spectrum of pure zeolite contained two additional bands at: 741.97 cm^−1^ and 658.57 cm^−1^, which can be attributed to symmetric T-O-T stretching and symmetric Si-O-Si stretching, respectively. All above bands are typical for zeolites X [38].

### 3.2. Zn^2+^ and Pb^2+^ Adsorption on Zeolite and Zeolite–Carbon Composite in the Single Systems

**Figure 6:**
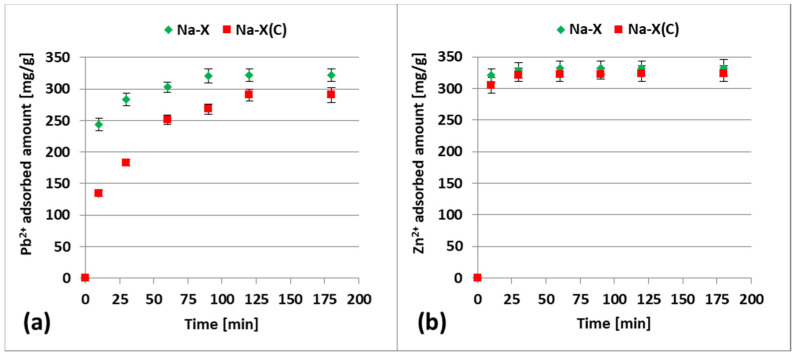
Adsorption kinetics of Pb^2+^ (**a**) and Zn^2+^ (**b**) on the Na-X and Na-X(C) surface in the single systems, at pH 5.

Figure 6a,b present the measured adsorbed amounts of heavy metal ions on zeolite and zeolite–carbon composite over time.

Experimental data were fitted to the commonly applied kinetic models—the pseudo-first-order and pseudo-second-order equations. The calculated parameters indicated the best fitting of experimental data to the pseudo-second-order model. The R^2^ values were as follows: 0.999 (Na-X + Pb^2+^), 0.997 (Na-X(C) + Pb^2+^), 1.000 (Na-X + Zn^2+^) and Na-X(C) + Zn^2+^; Table 3).

According to the literature [39,40], a good fitting of experimental data to the PSO model indicated that the adsorption process was based on chemical interactions (chemisorption). The valence forces through the exchange or sharing of electrons between adsorbate and adsorbent. Such a good fitting of experimental data to PSO was also observed during the study on lead(II), chromium(VI) and copper(II) adsorption on kaolinite [41,42,43], as well as copper(II) and silver(I) ions on biochar [44,45]. However, it must be emphasized that some researchers do not agree with these assumptions. Płaziński and Rudziński [46] stated that the PSO/PFO models are not able to reflect changes in the mechanism controlling the adsorption kinetics. Thus, these equations do not represent any specific physical model. Qiu et al. [47] wrote that in many cases kinetic models are applied in an unsuitable or improper manner due to their boundary conditions.

The obtained results showed that in the single systems (containing only one heavy metal) the equilibrium state was reached faster for Zn^2+^ ions than for Pb^2+^—120 min for Pb^2+^ and 10 min for Zn^2+^. After these times, the amount of the ions adsorbed on the solid surface does not change over time. The calculated k_2_ values for Zn^2+^ adsorption was significantly higher (compared to Pb^2+^) and were in the range 0.0069–0.0090 g/mg·min.

**Figure 7:**
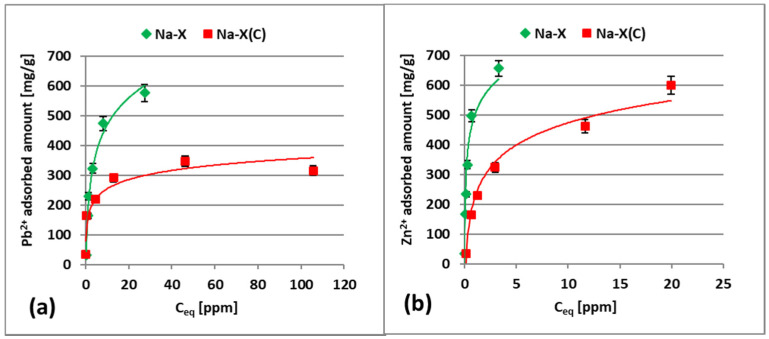
Adsorption isotherms of Pb^2+^ (**a**) and Zn^2+^ ions (**b**) on the Na-X and Na-X(C) surfaces in the single systems at pH 5.

Figure 7a,b show the experimental adsorption isotherms for Zn^2+^ and Pb^2+^ ions on the Na-X and Na-X(C) surfaces in the single systems.

The isotherm parameters calculated using Langmuir and Freundlich models are shown in Table 4.

The experimental data were better fitted to the Langmuir model. The R^2^ values for all systems were very high and equal to 0.966 (Na-X + Pb^2+^), 0.997 (Na-X(C) + Pb^2+^ and Na-X + Zn^2+^) and 0.979 (Na-X(C) + Zn^2+^). Therefore, it can be assumed that Pb^2+^ and Zn^2+^ form monolayers on the Na-X and Na-X(C) surfaces and all adsorption sites are energetically equivalent [27,40,48]. A good fitting of experimental data to the Langmuir model was also observed for the systems: kaolinite-Pb^2+^, kaolinite-Cr(VI), kaolinite-Cu^2+^ and activated biochar-Cu^2+^ [41,42,43,45]. The obtained data also indicated that larger amounts of metal ions were adsorbed on the zeolite than on its carbon composite. The q_m_ parameter (indicating maximum adsorption capacity in Langmuir model) of zeolite relative to Pb^2+^ ions equal 676.59 mg/g, whereas zeolite relative to Zn^2+^ ions equal 321.91 mg/g. On the other hand, the q_m_ value of zeolite–carbon composite was 693.29 mg/g and 640.94 mg/g relative to Pb^2+^ and Zn^2+^, respectively. The higher adsorption capacity of zeolite (compared to zeolite–carbon composite) is mainly dictated by its larger specific surface area. The S_BET_ parameter of Na-X is 456 m^2^/g higher than that of Na-X(C). Moreover, pure zeolite is characterized by higher volume of micropores (V_micro_ is equal to 0.27 cm^3^/g and 0.07 cm^3^/g for Na-X and Na-X(C), respectively).

The adsorbed amounts of Zn^2+^ observed for both adsorbents were significantly higher than those of Pb^2+^. It can be explained by differences in the ion sizes—zinc has a much smaller ionic radius (0.74 Å) than lead (1.29 Å) [49,50]. Owing to the smaller size, Zn^2+^ ions reach active sites of adsorbent faster. During the adsorption, cations located in zeolite pores can be replaced with heavy metal ions. This ion exchange is easier for Zn^2+^ ions and occurs according to equation (Equation (5)) [50]:

Zn^2+^_(solution)_ + 2Na^+^_(zeolite)_ ⇌ Zn_(zeolite)_ + 2Na^+^_(solution)_ (5)

The point of zero charge (pH_pzc_) of zeolite is about 9, whereas of Na-X(C) is 8.5. At pH values lower than pH_pzc_, the adsorbent surface has a positive charge, whereas at pH values higher than pH_pzc_ the adsorbent surface assumes a negative charge. Thus at pH 5, when both adsorbent and adsorbate have a charge of the same sign, there is an electrostatic repulsion between the system compounds. However, despite the presence of these forces, heavy metal ions can be adsorb from the solution efficiently. This is probably dictated by the large specific surface area of the solids and their unique structure composed of three-dimensional channels and pore system allowing exchange of ions [1,2].

### 3.3. Zn^2+^ and Pb^2+^ Adsorption on Zeolite and its Composite in the Mixed Systems

**Figure 8:**
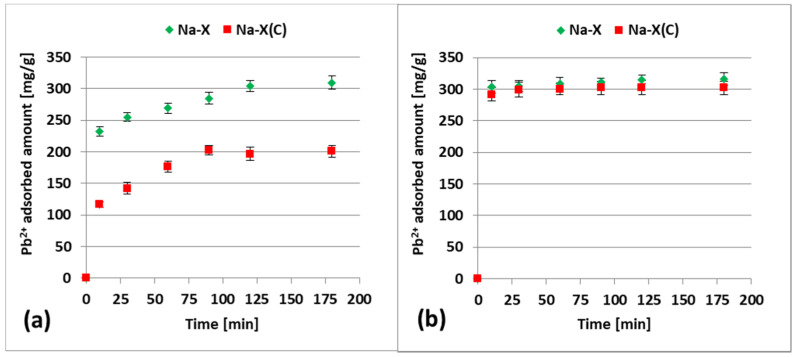
Adsorption kinetics of Pb^2+^ (**a**) and Zn^2+^ (**b**) on Na-X and Na-X(C) in the mixed systems, at pH 5.

**Figure 9:**
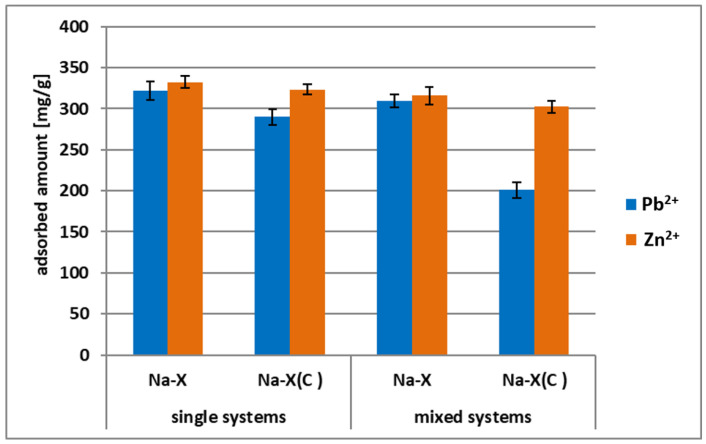
Comparison of adsorbed amounts of Pb^2+^ and Zn^2+^ on Na-X and Na-X(C) in the mixed systems, in an equilibrium state at pH 5.

Figure 8a,b present adsorption kinetics of Zn^2+^ and Pb^2+^ on Na-X and Na-X(C) in the mixed systems (i.e., containing both heavy metals at the same time). In turn, Figure 9 summarizes the adsorbed amounts of heavy metals on both adsorbents noted in the single and mixed systems.

The obtained data indicated that the adsorbed amounts observed in the mixed systems were clearly lower than those noted in the single ones. The differences were noticeable for both heavy metal ions. In case of Pb^2+^ ions, the adsorbed amount on the Na-X surface decreased from 322.1 mg/g to 309.36 mg/g, whereas on the Na-X(C) surface, it decreased from 290.11 mg/g to 200.76 mg/g. In the case of Zn^2+^ ions, the adsorbed amount on the Na-X surface decreased from 332.506 mg/g to 315.74 mg/g, in turn on the Na-X(C) surface it decreased from 323.53 mg/g to 302.56 mg/g. Thus, there is a competition between Zn^2+^ and Pb^2+^ ions for the adsorbent active sites in the mixed systems. However, it must be emphasized that the simultaneous addition of two ions to the system did not significantly affect the time needed to reach equilibrium. These times, noted in the mixed solutions, were identical to those observed in the single ones and equaled 120 min for Pb^2+^ ions and 10 min for Zn^2+^ ions.

### 3.4. Impact of Zn^2+^/Pb^2+^ Adsorption on Surface Charge Density of Na-X and Na-X(C) Particles

**Figure 10:**
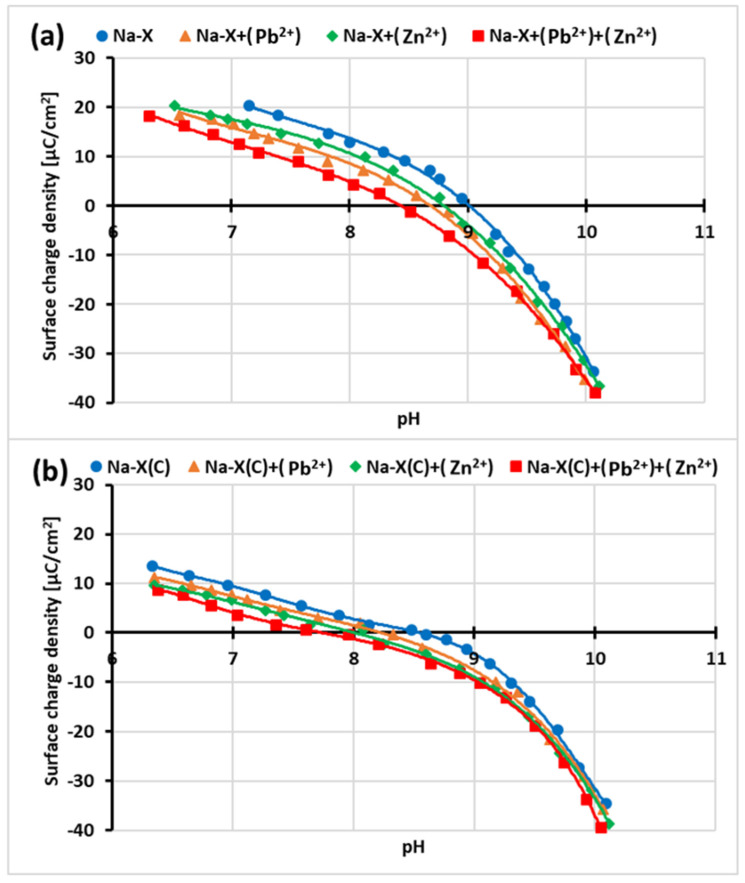
Surface charge density vs. solution pH of Na-X (**a**) and Na-X(C) (**b**) with and without one or two heavy metal ions.

Figure 10a,b present changes in the surface charge density (σ_0_) of Na-X and Na-X(C) materials as a function of the solution pH.

The obtained titration curves indicated that at pH 5, at which the adsorption measurements were performed, both Na-X and Na-X(C) have a positively charged surface. Thus the repulsion between surface groups and heavy metal occurs at pH 5. Despite these unfavorable electrostatic conditions, adsorption of Pb^2+^ and Zn^2+^ takes place through chemical interactions (which was discussed above). The point of zero charge (pH_pzc_) for Na-X is about 9, whereas for Na-X(C) it is 8.5. The pH_pzc_ falls at pH value for which the concentrations of the positively and negatively charged surface groups are the same (i.e., σ_0_ = 0). For both adsorbents, the addition of heavy metal ions causes a decrease in pH_pzc_ values. The pH_pzc_ changes of Na-X particles are noticeable when Zn^2+^ and Pb^2+^ are added—their values decrease to about 8.8 and 8.6, respectively. The addition of both ions simultaneously causes further decrease of pH_pzc_ to 8.5. When Pb^2+^ and Zn^2+^ are added to the system separately containing Na-X(C), a decrease in pH_pzc_ values to 8.3 and 8.1 was observed. In turn, the addition of both ions lowers the pH_pzc_ to 7.8.

The addition of divalent cations caused a decrease in the pH_pzc_ value of both the zeolite and its composite with carbon (Equations (6–11)). It is related to the decrease in the value of the surface charge density due to the formation of an additional number of negatively charged groups on the adsorbent surface. This phenomenon results from the interaction of metal cations with the solid surface groups, according to the equations [51,52]:

−SOH + Pb^2+^ ⇌ −SO^−^Pb^2+^ + H^+^ (6)

2(−SOH) + Pb^2+^ ⇌ (−SO^−^)_2_Pb^2+^ + 2H^+^ (7)

−SOH + Pb^2+^ + H_2_O ⇌ − SO^−^PbOH^+^ + 2H^+^ (8)

−SOH + Zn^2+^ ⇌ − SO^−^Zn^2+^ + H^+^ (9)

2(−SOH) + Zn^2+^ ⇌ (−SO^−^)_2_Zn^2+^ + 2H^+^ (10)

−SOH + Zn^2+^ + H_2_O ⇌ −SO^−^ZnOH^+^ + 2H^+^ (11)

Simple ion adsorption affects usually the surface charge of adsorbents. It was described, inter alia, by Fijałkowska et al. [41,53,54,55]. In their studies, Pb^2+^ ions caused an increase in the negative values of σ_0_ of two clay minerals, kaolinite and montmorillonite. A similar behavior was observed for the Cu^2+^ ions adsorption on the carbon–silica composite surface [56,57].

### 3.5. Impact of Zn^2+^/Pb^2+^ Adsorption on Zeta Potential of Na-X and Na-X(C) Particles

**Figure 11:**
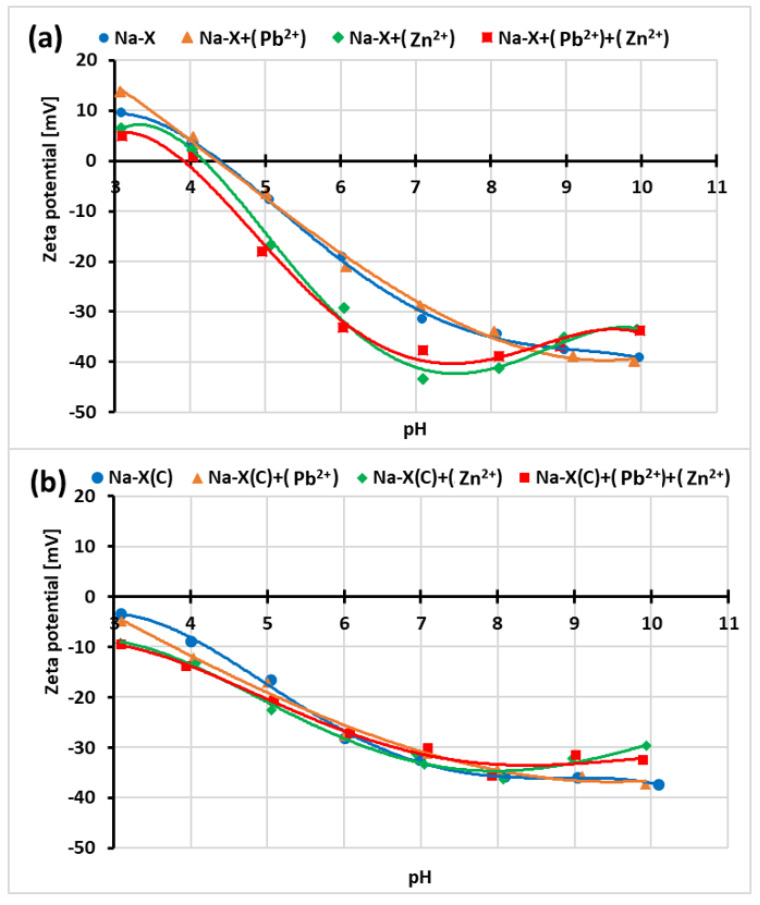
Zeta potential vs. solution pH of Na-X (**a**) and Na-X(C) (**b**) particles with and without one or two heavy metal ions.

Figure 11a,b present the pH effect on the zeta potential of absorbent particles.

The isoelectric point (pH_iep_) of the zeolite particles occurs at about pH 4.5. In the case of Na-X(C) particles, pH_iep_ is not observed in the studied pH range. The iep is at pH value for which the concentrations of the positively and negatively charged ions in the slipping plane area are the same (i.e., ζ = 0). At a pH below pH_iep_, the zeta potential becomes positive due to the predominance of positively charged ions in the slipping plane area. On the other hand, at pH below pH_iep_, this trend is opposite (negatively charged ions dominate in the slipping plane area). Moreover, the iep value for zeolite materials without adsorbates are significantly different from the pzc values. This is mainly due to the overlapping of the diffusion parts of the electrical double layer formed on the opposite walls inside the relatively small solid pores. Such behavior was also observed for suspensions containing other zeolites [58,59,60].

The changes in the pH_iep_ of Na-X particles are noticeable when Zn^2+^ and both cations are added, the pH_iep_ values decrease to about 4.1 and 3.9, respectively. The addition of Pb^2+^ does not significantly affect this parameter. In the case of Na-X(C), the addition of ions practically does not change the zeta potential of solid particles. At pHs equal or higher than 8.5, there is a trend of decrease in the zeta potential with pH increase. It is observed especially in single systems containing Zn^2+^ ions. This is due to overcharging or overloading of the electrical double layer, i.e., charge reversal. This phenomenon is related to the occurrence of more numerous ions on the inner part of the electrical double layer than the ions on the solid surface. Consequently, the same charge sign appears on the outside part of the electrical double layer and also on the surface [61].

The zeta potential decides the stability of solid particles covered with adsorption layers. This parameter is very important for the effective separation of the solid with adsorbed heavy metals from the liquid phase. In the examined system, zeta potential values in all examined systems at pH 5 (at which adsorption of heavy metals was performed) changes from −5 to −22 mV. Such values of electrokinetic potential do not guarantee effective suspensions stabilization. According to the literature [62], the colloidal system is stable when absolute values of zeta potential of the particles exceed 30 mV.

### 3.6. Desorption of Zn^2+^ and Pb^2+^ from Zeolite and Its Composite

The desorption degrees observed for the solids are shown in Table 5.

These results indicated significantly larger desorption using 0.1 M HCl than 0.1 M NaOH. Furthermore, the measured desorbed amounts were higher for Pb^2+^ ions than Zn^2+^ ones. These trends were observed for both Na-X and Na-X(C), in the single and mixed systems.

The higher desorption of Pb^2+^ ions is caused by the fact that during the adsorption Zn^2+^ cations are exchanged more effectively with the ions located in the structure of zeolite or zeolite–carbon composite. Pb^2+^ ions, owing to their larger size, do not penetrate the pores and channels in such a great degree as Zn^2+^ ones, so their desorption is easier.

## 4. Conclusions

In this work, Na-X zeolites and a carbon zeolite composite Na-X(C) were used to remove Zn^2+^ and Pb^2+^ (single and mixed systems) from aqueous solutions. Pure zeolite was characterized by a more developed surface than zeolite–carbon composite. The adsorption of Zn^2+^ ions occurs very quickly and efficiently, which is evidenced by the achievement of equilibrium in both systems (Zn^2+^ + Na-X/Na-X(C)) just after 10 min. Due to the larger specific surface area of Na-X, the ion adsorption is more efficient on its particles than on the Na-X(C) ones. Experimental data of adsorption kinetics were the best fitted to the pseudo-second-order model. In turn, the obtained adsorption isotherms were better fitted to the Langmuir model. In the mixed systems, there is a competition between the heavy metal ions, which leads to reduced adsorption of Pb^2+^ and Zn^2+^. The addition of divalent cations decreases the pH_pzc_ of the studied systems due to the induction of additive negative sites on the adsorbent surface. More efficient desorption of heavy metals from the solids was achieved using hydrochloric acid. Less efficient desorption of Zn^2+^ ions indicates a stronger binding of this ion to the surface of adsorbents. The novelty of the research is the fact that a new way to dispose of fly ash has was successfully discovered. A normal use of such waste (e.g., in construction industry), is not possible due to the high carbon content. Using the secondary waste (silica and alumina rich solution) for the production of pure zeolite (Na-X) is an another innovative aspect of this study. These facts, combined with the possibility of producing adsorbents on a semi-technical scale, will allow to reduce the amount of disposed fly ash, and contribute to more efficient heavy metals removal from aqueous solutions.

## Figures and Tables

**Table 1:** Textural parameters of Na-X and Na-X(C) materials: S_BET_—specific surface area, S_micro_—micropore area, V_t_—total pore volume, V_micro_—micropore volume, D—average pore diameter.

Material	S_BET_ (m^2^/g)	S_micro_ (m^2^/g)	V_t_ (cm^3^/g)	V_micro_ (cm^3^/g)	D (4V/A) (nm)
Na-X	728	694	0.31	0.27	1.73
Na-X(C)	272	189	0.17	0.07	2.56

**Table 2:** Elemental composition of Na-X(C) and Na-X adsorbents (%).

Adsorbent	Compounds											
	Na_2_O	MgO	Al_2_O_3_	SiO_2_	P_2_O_5_	SO_3_	K_2_O	CaO	TiO_2_	Fe_2_O_3_	LOI	C
Na-X	7.32	1.69	23.41	41.12	0.02	-	0.71	7.48	0.05	1.31	16.88	-
Na-X(C)	3.53	1.21	14.37	28.19	0.05	0.48	1.34	2.21	1.05	9.33	38.23	31.46

**Table 3:** Kinetics parameters of Pb^2+^ and Zn^2+^ adsorption on Na-X and Na-X(C) at pH 5.

		Pseudo-II-Order Model			
System		q_e_ (mg/g)	k_2_ (g/(mg·min))	R^2^	Linear Form
Pb^2+^	Na-X	333.333	0.0007	0.999	y = 0.003x + 0.0129
	Na-X(C)	322.581	0.0002	0.997	y = 0.0031x + 0.0533
Zn^2+^	Na-X	333.333	0.0090	1.000	y = 0.003x + 0.001
	Na-X(C)	322.581	0.0069	1.000	y = 0.0031x + 0.0014

**Table 4:** Isotherm parameters of Pb^2+^ and Zn^2+^ adsorption on the Na-X and Na-X(C) surfaces at pH 5.

		Langmuir Model			Freundlich Model		
System		q_m_ (mg/g)	K_L_ (dm^3^/mg)	R^2^	n	K_F_ (mg/g (mg/dm^3^)^−1/nF^)	R^2^
Pb^2+^	Na-X	676.594	0.227	0.966	1.625	0.0005	0.794
	Na-X(C)	321.909	1.178	0.997	2.501	0.0014	0.719
Zn^2+^	Na-X	693.289	4.878	0.997	2.148	0.0002	0.943
	Na-X(C)	640.938	0.395	0.979	1.899	0.0006	0.916

**Table 5:** Desorption degree (%) of Zn^2+^ and Pb^2+^ ions from the Na-X and Na-X(C) surface in single and mixed systems, obtained using 0.1 M HCl or 0.1 M NaOH.

Desorbing Agent	Desorbed Ion	Na-X	Na-X(C)
		Single Systems	
HCl	Pb^2+^	61.00 ± 1.5	39.55 ± 1.6
NaOH		17.13 ± 0.6	12.29 ± 0.4
HCl	Zn^2+^	6.11 ± 0.1	3.82 ± 0.2
NaOH		2.48 ± 0.1	1.32 ± 0.1
		**Mixed Systems**	
HCl	Pb^2+^	77.94 ± 2.1	64.31 ± 1.8
NaOH		24.82 ± 1.2	22.33 ± 1.5
HCl	Zn^2+^	6.43 ± 0.2	4.08 ± 0.1
NaOH		2.61 ± 0.1	1.41 ± 0.1

## Data Availability

The data presented in this study are available on request from the corresponding author.
